# Arrearages

**Published:** 1868-08-01

**Authors:** 


### *Arrearages.*

Subscribers who have allowed themselves to get in arrears for the JOURNAL are particularly notified that its business matters have, since January last, been placed in the hands of our thoroughly competent and accurate publisher, who means to conduct every thing in a business way. The editor has not, and will not write dunning letters. But the publisher has no respect of persons, and proposes to keep harping on the business strings until the JOURNAL secures that ample pecuniary independence it has fairly earned. It is our intention to publish *weekly*, from and after next January, which will necessarily involve a large increase in expenditure. We propose to fortify in season. "Short accounts make long friends." There is not, we repeat, the slightest occasion for those who are dunned to write the editor sharp, smart or lugubrious replies. They only help to fill the waste basket.

The price of the JOURNAL, as has been advertised in every number, is THREE DOLLARS *per annum* in advance--if not paid within six months, FOUR DOLLARS. Any publisher will inform subscribers that the advance payment is worth much more to him than the delayed one, even at the increased price. To encourage in well-doing, however, we propose to those still in arrears to abate the extra dollar on subscription if the account is settled within thirty days from the receipt of this number of the JOURNAL. All mistakes cheerfully rectified.

